# Highly Accelerated Real-time Cine MRI Pulse Sequence for Cardiac Implantable Electronic Devices and Arrhythmias

**DOI:** 10.1148/ryct.240554

**Published:** 2025-11-13

**Authors:** Dima Bishara, Kyungpyo Hong, María Davó-Jiménez, Jeremy D. Collins, Cagdas Topel, Lexiaozi Fan, Roberto Sarnari, Bradley K. Knight, Daniel C. Lee, Daniel Kim

**Affiliations:** ^1^Department of Radiology, Northwestern University Feinberg School of Medicine, 737 N Michigan Ave, Ste 1600, Chicago, IL 60611; ^2^Department of Biomedical Engineering, McCormick School of Engineering, Northwestern University, Evanston, Ill; ^3^Department of Medicine (Cardiology), Northwestern University Feinberg School of Medicine, Chicago, Ill; ^4^Department of Radiology, Mayo Clinic, Rochester, Minn; ^5^Division of Cardiology, Internal Medicine, Northwestern University Feinberg School of Medicine, Chicago, Ill

**Keywords:** MR Imaging, Cardiac, Heart, Left Ventricle, Right Ventricle, Artifacts, Cardiac Assist Devices, Imaging Sequences, Radial K-Space, CIED, Arrhythmia, GRASP, Compressed Sensing, Real-Time, Freebreathing

## Abstract

**Purpose:**

To compare the image quality and temporal resolution of a 32-fold accelerated real-time cine MRI pulse sequence (19.3-msec temporal resolution) with those of a previously described 16-fold accelerated Cartesian real-time cine sequence (39.8-msec temporal resolution) in patients with cardiac implantable electronic devices (CIEDs) and arrhythmias.

**Materials and Methods:**

This retrospective study included patients with CIEDs and arrhythmias scanned using both radial and Cartesian real-time cine MRI pulse sequences with gradient-recalled echo readouts at 1.5 T from March 2022 to March 2024. Image quality was assessed visually by clinical raters using a five-point Likert scale for each of four categories (namely, conspicuity, temporal fidelity of wall motion, artifact, and noise) and quantitatively using the signal-to-noise-and-artifact ratio metric. The authors used the Wilcoxon signed rank test and paired *t* test to compare differences for non-normally and normally distributed variables, respectively.

**Results:**

The study included 23 patients with CIEDs and arrhythmias (nine female and 14 male patients; mean age, 66.5 years ± 13.3 [SD]). The radial real-time cine sequence yielded significantly higher median summed visual scores than the Cartesian real-time cine sequence across all myocardial segments (radial, 17.5–18, Cartesian, 14.5–16; *P* < .001). The mean signal-to-noise-and-artifact ratio was significantly higher for radial than for Cartesian (radial, 18.3 ± 5.6, Cartesian, 9.4 ± 4.1; *P* < .001).

**Conclusion:**

The proposed 32-fold accelerated radial real-time cine MRI pulse sequence provided higher temporal resolution and superior image quality than the 16-fold accelerated Cartesian real-time cine pulse sequence in patients with CIEDs and arrhythmias.

**Keywords:** MR Imaging, Cardiac, Heart, Left Ventricle, Right Ventricle, Artifacts, Cardiac Assist Devices, Imaging Sequences, Radial K-Space, CIED, Arrhythmia, GRASP, Compressed Sensing, Real-Time, Freebreathing

*Supplemental material is available for this article.*

© The Author(s) 2025. Published by the Radiological Society of North America under a CC BY 4.0 license.

**Summary**

A 32-fold accelerated real-time cine MRI sequence (radial sampling) demonstrated better temporal resolution and image quality than a 16-fold sequence (Cartesian sampling) in patients with cardiac implantable electronic devices and arrhythmias.

**Key Points**

■ In this retrospective study, 23 patients with cardiac implantable electronic devices (CIEDs) and arrhythmia underwent 1.5-T real-time cine MRI using a 32-fold accelerated gradient-echo sequence.
■ View-sharing and k-space–weighted image contrast filtering in golden-angle radial MRI achieved a 32-fold acceleration and 19.3-msec temporal resolution, enabling clinically acceptable image quality.
■ The 32-fold accelerated radial cine MRI sequence provided higher temporal resolution (19.3 msec vs 39.8 msec) and summed visual scores (17.5 [IQR, 1.5] vs 15 [IQR, 2], *P* < .0001) than a 16-fold accelerated Cartesian cine MRI sequence.

## Introduction

Cardiac cine MRI is the reference imaging test for assessing biventricular volumes and functional parameters (1–3). However, in patients with cardiac implantable electronic devices (CIEDs) (4), several technical challenges compromise image quality (5). First, the CIED generator produces kilohertz-range variations in the static magnetic field across the heart (6,7). These field distortions preclude the use of balanced steady-state free precession readouts, particularly in patients with implantable cardioverter defibrillators (8). For both MR safety and image artifact considerations, patients with CIEDs should be scanned using gradient-recalled echo readouts with low flip angles (ie, reduced radiofrequency-induced heating) instead (9). Second, patients with a CIED often have a high arrhythmia burden, prompting the CIED to manage the underlying rhythm disorder (10). In patients with a high arrhythmia burden, a standard retrospective electrocardiographically (ECG) gated cine pulse sequence is likely to produce severe ghosting and blurring artifacts, resulting in blurred blood-to-myocardial boundaries (11,12). Third, repeated imaging because of poor performance with ECG-gated cine scans lengthens total examination time, increasing both MR safety risks and patient discomfort. The results of multiple studies have demonstrated that real-time cine imaging with balanced steady-state free precession readout offers advantages over ECG-gated breath-hold balanced steady-state free precession cine imaging in patients with arrhythmia and no CIEDs (13–17); however, the performance of real-time cine imaging using gradient-recalled echo readouts in patients with arrhythmia and CIEDs remains unexplored.

Although a previously described 16-fold accelerated real-time cine pulse sequence using Cartesian k-space sampling and compressed sensing (18) demonstrated diagnostically acceptable image quality and reasonably accurate left ventricular volumes and functional parameters in patients with CIED with sinus rhythm (19), its performance specifically in patients with CIEDs and arrhythmias remains untested. A potential limitation of the 16-fold accelerated Cartesian real-time cine imaging is its marginally acceptable temporal resolution of 40 msec (20), which may be insufficient for patients with CIEDs with a high burden of arrhythmia and/or tachycardia. In these patients, higher temporal resolution may be clinically advantageous for mitigating the impact of temporal wall motion blurring arising from over-regularization in compressed sensing or golden-angle radial sparse parallel reconstruction (21). One approach to accelerate the pulse sequence and increase the temporal resolution is to use radial k-space sampling with tiny golden angles (22) and amplify it using view sharing (23,24) and k-space–weighted image contrast filtering (25), as previously introduced with balanced steady-state free precession readouts for patients without CIEDs (26).

This study aimed to compare the performance of a 32-fold accelerated radial real-time cine pulse sequence using gradient-recalled echo readouts and the previously introduced 16-fold accelerated Cartesian real-time cine pulse sequence using gradient-recalled echo readouts exclusively for patients with CIEDs and arrhythmias.

## Materials and Methods

This retrospective study was conducted according to protocols approved by our institutional review board and complied with the Health Insurance Portability and Accountability Act. All patients provided written informed consent and agreed to participate in the parent research study. Data generated or analyzed during the study are available from the corresponding author upon request and establishment of a data sharing agreement.

### Study Sample

The study included patients with CIEDs who were scanned using real-time cine pulse sequences from March 2022 to March 2024. The patients were screened for a prior history of atrial fibrillation and the presence of arrhythmia during MRI, defined by the “ECG” trace recorded in the raw k-space data header. Because the ECG signal inside the MRI bore can be distorted because of magnetohydrodynamic effects (27), the exact type of arrhythmia was not diagnosed during the MRI procedure. Arrhythmia was defined by heart rate variability, calculated as the ratio of the IQR of the R-R intervals to the median R-R intervals, using a cutoff value greater than 10% (28).

### Imaging Procedures

Patients were scanned with one 1.5-T MRI scanner (Avanto; Siemens Healthineers) equipped with a gradient system capable of achieving a maximum gradient strength of 45 mT/m and a maximum slew rate of 200 T/m/sec. A body coil was used for radiofrequency excitation, and standard body flex and spine coil arrays (12–15 elements) were used for signal reception.

Both real-time cine pulse sequences were performed during free breathing to sample the whole left ventricle in the short-axis orientation. Table 1 summarizes the relevant imaging parameters for both real-time cine pulse sequences. Both cine pulse sequences used gradient-recalled echo readouts to minimize off-resonance effects caused by the CIED generator. The pulse sequence order was randomized to minimize potential bias.

**Table 1:** Key Imaging Parameters of Cartesian Cine and Free-breathing Real-time Radial Cine Sequences

Pulse Sequence Parameters	Cartesian	Radial
FOV (mm^2^)	400 × 400	400 × 400
Section thickness (mm)	8	8
Reconstruction matrix	192 × 192	192 × 192
Spatial resolution, in-plane (mm^2^)	2.1	2.1
TR/TE (msec)	3.06/1.5	3.2/1.41
Flip angle (°)	20	20
Receiver bandwidth (Hz/pixel)	745	745
K-space trajectory	Cartesian	Radial
Reordering	Linear	32.0397° increment
Readout	GRE	GRE
Acceleration factor	14.8 (13 k-space lines per frame)	32 (6 k-space lines per frame)
Temporal resolution (msec)	39.8	19.3
No. of cardiac phases	138	249
Scan duration (sec)	5	5

Note.—FOV = field of view, GRE = gradient-recalled echo, TE = echo time, TR = repetition time.

### Image Reconstruction

**Figure 1:**
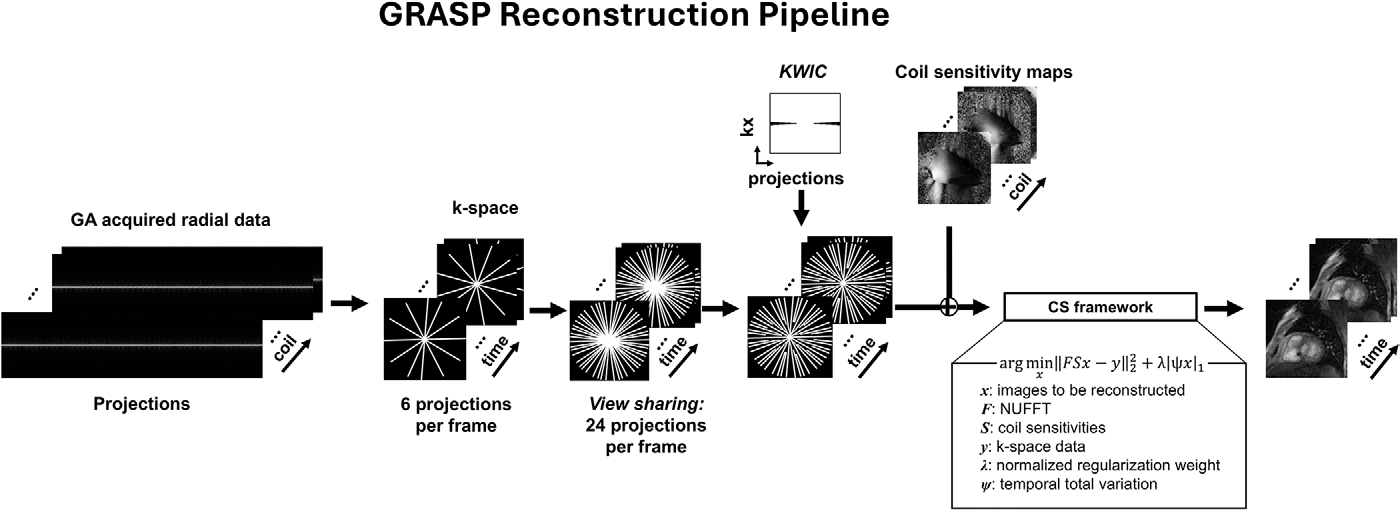
The GRASP reconstruction pipeline incorporating VS and KWIC filtering. The VS scheme consists of six native projections per frame with nine projections before and nine projections after, for a total of 24 k-space projections per frame. KWIC filtering was used to remove the center of k-space of shared k-space lines. GRASP reconstruction details included the following: sparsifying transform = temporal total variation, number of iterations = 36, normalized regularization weights = 0.001. CS = compressed sensing, GA = golden angle, GRASP = golden-angle radial sparse parallel, KWIC = k-space–weighted image contrast, NUFFT = nonuniform fast-Fourier transform, VS = view-sharing.

We performed custom-made golden-angle radial sparse parallel and compressed sensing image reconstructions on a graphics processing unit workstation (32 Xeon E5-2620 v4, 384-GB memory; Intel) equipped with two graphics processing unit cards (Tesla V100 graphics processing unit with 32-GB memory; NVIDIA), running on a Linux operating system (Ubuntu 24.04.2 LTS). Our custom-made reconstruction codes were written using MATLAB (version R2023b; The MathWorks). Cartesian real-time cine data were reconstructed using compressed sensing, as previously described (19). Radial real-time cine data were reconstructed using golden-angle radial sparse parallel, as previously described (26) and illustrated in Figure 1. While preserving the overall image reconstruction scheme (26), we implemented two key modifications: *(a)* Each cardiac frame was reconstructed with six native radial spokes supplemented by 18 shared spokes (nine before and nine after) and *(b)* a triangular-shaped k-space–weighted image contrast filter was employed to remove the centers of the shared k-space lines to suppress temporal blurring associated with k-space sharing.

### Visual Assessment of Image Quality

The resulting real-time cine images were randomized and de-identified for independent visual assessment by two attending noninvasive cardiologists (R.S. with 6 years and M.D.J. with 1 year of experience) using a five-point Likert scale across four categories for all 16 myocardial segments. These categories included *(a)* conspicuity of the endocardial border (reflecting spatial resolution and image contrast), *(b)* temporal fidelity of wall motion (reflecting temporal resolution), *(c)* noise levels, and *(d)* artifact levels. For conspicuity and temporal fidelity, the assigned scores were 1 = nondiagnostic, 2 = poor, 3 = clinically acceptable, 4 = good, and 5 = excellent. For noise and artifact levels, the assigned scores were 1 = nondiagnostic, 2 = severe, 3 = moderate, 4 = mild, and 5 = minimal. The raters were masked to patient identity, pulse sequence, and other rater’s scores. Before visual scoring, training datasets were used to calibrate the cardiologists’ consensus scores. The summed visual score was defined as the numerical sum of all scores, and a summed visual score greater than 12 was defined as clinically acceptable. Based on a nonuniform distribution, the resulting visual scores were compared using the Wilcoxon signed rank test.

### Quantitative Analysis of Image Quality

**Figure 2:**
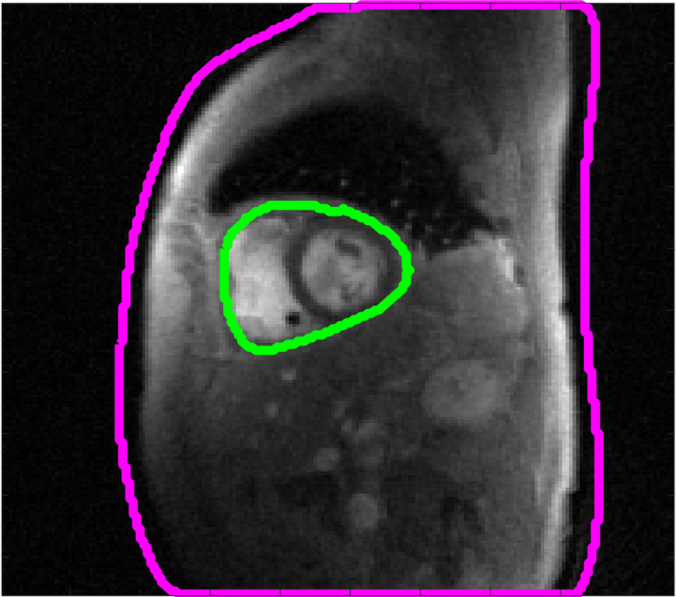
ROIs for SNAR calculation. Green ROI indicates the heart region, and the pink ROI indicates the foreground-background boundary. ROI = region of interest, SNAR = signal-to-noise-and-artifact ratio.

We calculated the signal-to-noise-and-artifact ratio to quantify the resulting image quality. The signal-to-noise-and-artifact ratio was calculated as the ratio of the mean signal intensity of the heart region (green area, Fig 2) to the SD of the background region (field of view excluding the purple area, Fig 2). The signal-to-noise-and-artifact ratio is similar to a signal-to-noise ratio but additionally accounts for artifacts present in the background.

### Statistical Analysis

Statistical analysis was conducted by one investigator (D.B.) using the Statistics and Machine Learning Toolbox in MATLAB (version R2023b). We used the Shapiro-Wilk test to assess the normality of variables. Matched variables between two groups were compared using two-tailed paired *t* test (Wilcoxon signed rank test, if not normally distributed). Average rater scores were used for comparisons across pulse sequences. Interrater reliability for ordinal data was calculated using the weighted Cohen κ test. *P* values less than .05 were considered statistically significant for each statistical test. Normally distributed data are presented as means ± SDs, whereas non-normally distributed data are presented as medians with IQRs in parentheses.

## Results

### Patient Characteristics

The study included 23 patients (mean age, 66.5 years ± 13.3; nine female, 14 male) with a CIED and a prior history of atrial fibrillation who were in arrhythmia during MRI as defined by the ECG trace recorded in the raw k-space data header (see Table 2 for patient demographics). All 23 patients successfully underwent both real-time cine pulse sequences. The scan time was five heartbeats per slice for each sequence. The mean heart rate variability was 14.0% ± 10.1.

**Table 2:** Patient Characteristics

Characteristic	Value
Age (y)	66.5 ± 13.3
Sex (male/female)	14/9 (60.9/39.1)
Smoking habit	11 (47.8)
Diabetes	13 (56.5)
Hypertension	16 (69.6)
eGFR (>90/>60/>15)	0/12/11 (0/52.2/47.8)
Heart rate (beats/min)	75.4 ± 12.52
HRV (%)	40.4 ± 24.4
LVEF (%)	47.0 ± 15.5
ICD	9 (39.1)
Pacemaker	13 (56.5)
CRT-D	4 (17.4)
CRT-P	0 (0)
S-ICD	0 (0)
History of myocardial infarction	8 (34.8)
History of revascularization	6 (26.1)
History of CAD	9 (39.1)
Atrial fibrillation	11 (47.8)
Stroke	4 (17.4)

Note.—Data are presented as means ± SDs or numbers, with percentages in parentheses. Heart rate variability (HRV) is calculated by dividing the IQR in R-R interval by the median of R-R interval during the cine scan. CAD = coronary artery disease, CRT-D = cardiac resynchronization therapy with defibrillator, CRT-P = cardiac resynchronization therapy with pacemaker, eGFR = estimated glomerular filtration rate, ICD = implantable cardioverter-defibrillator, LVEF = left ventricular ejection fraction, S-ICD = subcutaneous ICD.

### Representative Image Quality Comparison

**Figure 3:**
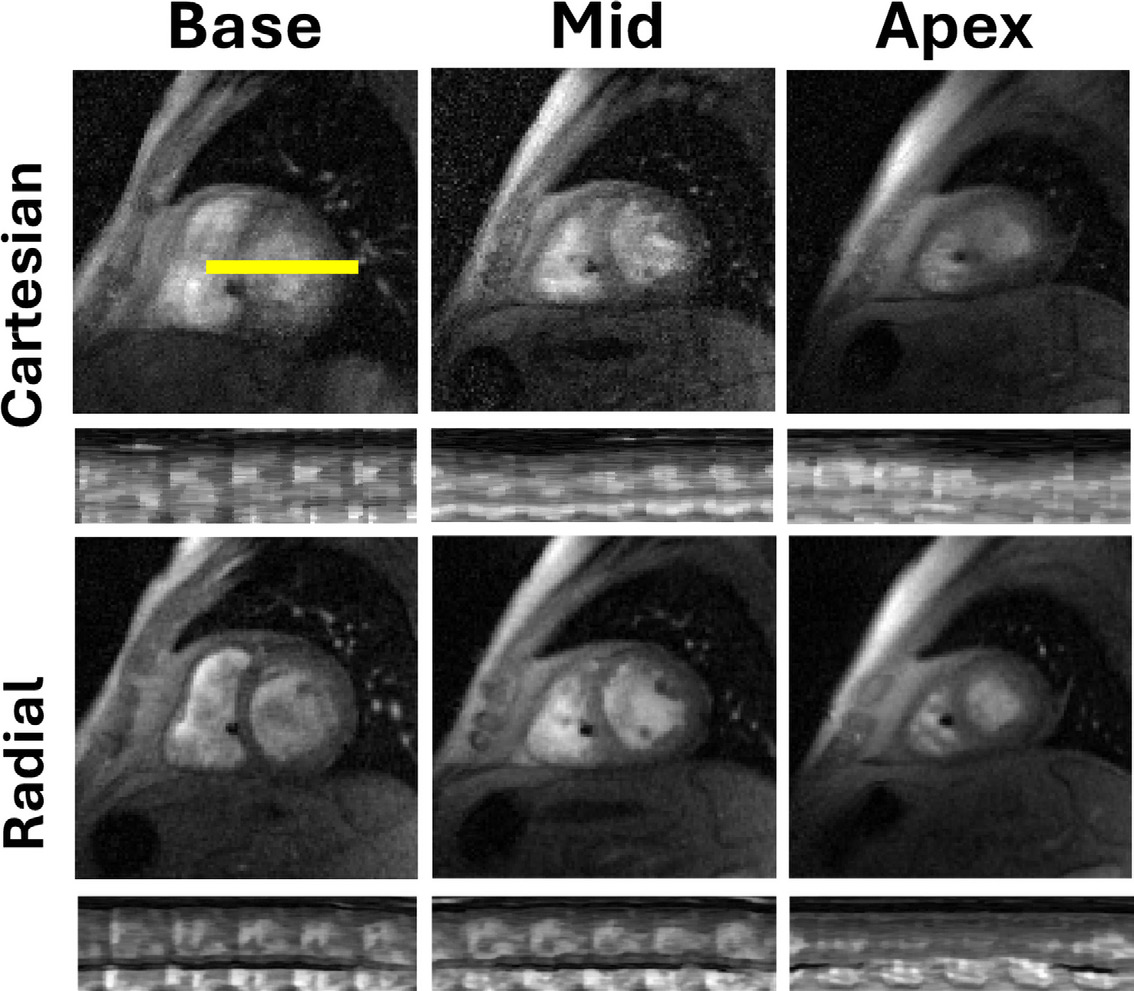
Cartesian (top row) and radial real-time cine (bottom row) MR images in one representative patient (64-year-old male patient) in three short-axis planes (base, mid, apex). Horizontal yellow line represents intensity-time profiles. For video display, see Movie 1 for Cartesian and Movie 2 for radial. For the “ECG” trace extracted from the raw data, please see Figure S1. ECG = electrocardiography.

Figure 3 compares radial and Cartesian real-time cine images in three short-axis planes for one patient with a CIED who had a heart rate variability of 20.7%. The intensity-time profiles shown reflect differences in temporal resolution between both cine sequences. For dynamic displays, see Movie 1 (Cartesian real-time cine) and Movie 2 (radial real-time cine). For the ECG trace derived from the metadata embedded in the raw k-space data, see Figure S1. As shown, radial real-time cine imaging produced better image quality than Cartesian real-time cine imaging.

**Movie 1:** Movie display of Cartesian real-time cine images in the patient shown in Figure 3.

**Movie 2:** Movie display of radial real-time cine images in the patient shown in Figure 3.

### Visual Score Assessment

**Figure 4:**
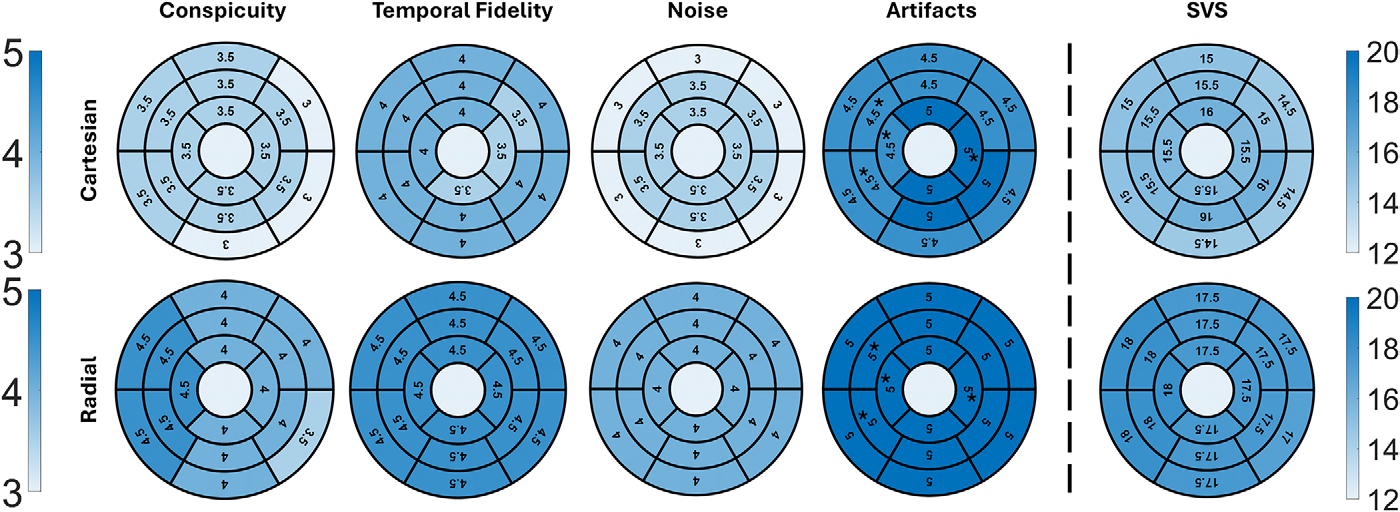
Bull’s-eye plots summarize the segmental visual scores in four categories: conspicuity of endocardial border, temporal fidelity of wall motion, noise, and artifacts. Each score ranges from 1 (worst) to 5 (best), with a score of 3 defined as clinically acceptable. SVS ranges from 4 (worst) to 20 (best), with a score of 12 defined as clinically acceptable. All segments showed significant differences between radial and Cartesian acquisitions, except for those marked with *. SVS = summed visual score.

Figure 4 shows bull’s-eye plots comparing segmental visual scores in four categories and summed visual scores between the two real-time cine pulse sequences. Although the median visual scores in all segments were above the clinically acceptable threshold for both sequences, differences were observed in conspicuity (radial ranging from 3.5 to 4.5, Cartesian, 3 to 3.5; *P* < .001), temporal fidelity of wall motion (radial, 4.5 to 4.5, Cartesian, 3.5 to 4; *P* = .001), noise (radial, 4 to 4, Cartesian, 3 to 3.5; *P* < .001), and summed visual scores (radial, 17 to 18, Cartesian, 14.5 to 16; *P* < .001). For artifacts, differences were observed in all segments (radial, 5 to 5, Cartesian, 4.5 to 5; *P* < .001), except in segments 8, 9, 14, and 16 (*P* > .05).

**Figure 5:**
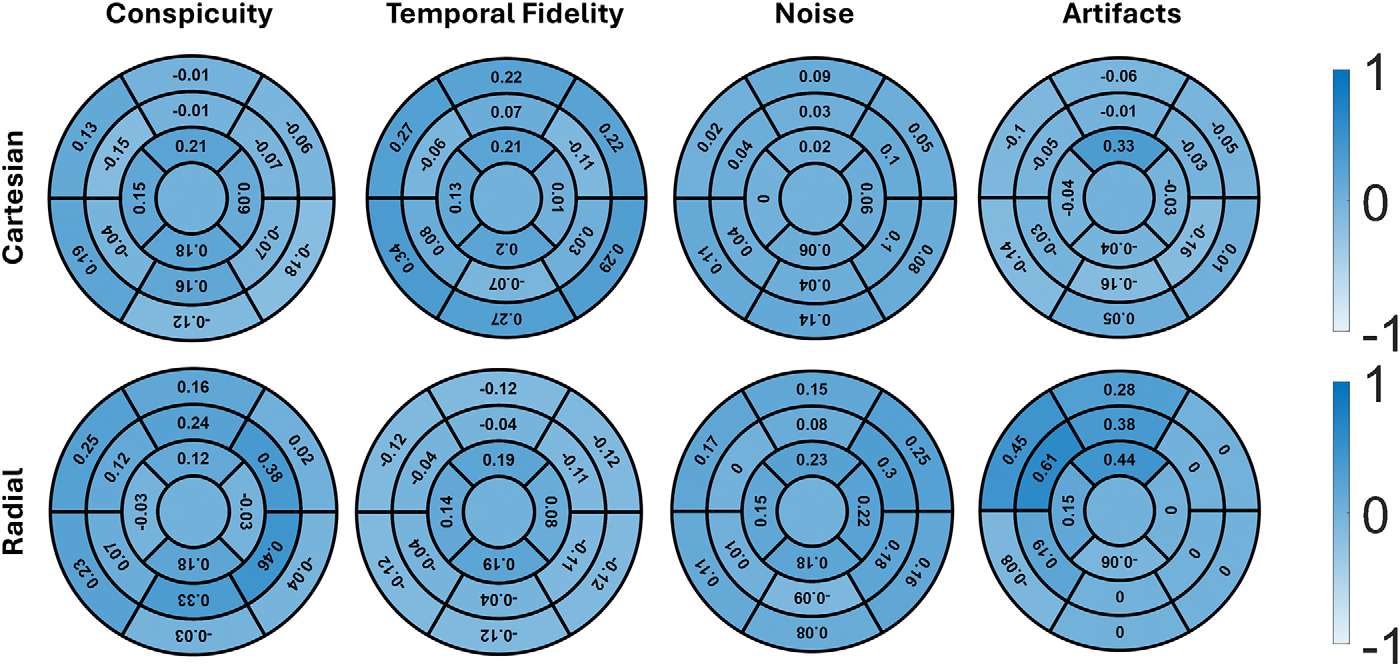
Bull’s-eye plots summarize interrater reliability in visual scoring across segments, using Cohen κ scores from −1 for poor agreement to +1 for perfect agreement.

Figure 5 shows bull’s-eye plots summarizing the segmental interrater reliability for visual scoring. Cohen κ values ranged from –0.12 (disagreement) to 0.61 (moderate agreement) for radial CT cines and from –0.18 (disagreement) to 0.34 (fair agreement) for Cartesian CT cines.

### Quantitative Analysis

The mean signal-to-noise-and-artifact ratio was higher for radial real-time cine than for Cartesian real-time cine imaging (radial, 18.3 ± 5.6, Cartesian, 9.4 ± 4.1; *P* < .001).

## Discussion

In this study, we compared the performance of a 32-fold, accelerated radial real-time cine MRI pulse sequence with that of a previously described 16-fold accelerated Cartesian real-time cine MRI pulse sequence in patients with CIEDs and arrhythmias. Radial real-time cine imaging produced better image quality, both in qualitative (radial summed visual scores = 17.5 [IQR, 1.5], Cartesian summed visual scores = 15 [IQR, 2]; *P* < .001) and quantitative (radial signal-to-noise-and-artifact ratio = 18.3 ± 5.6, Cartesian signal-to-noise-and-artifact ratio = 9.4 ± 4.1; *P* < .001) terms, than Cartesian real-time cine imaging.

Previous findings demonstrated that real-time cine imaging, with a temporal resolution ranging from 33 to 49 msec, provided superior image quality (ie, fewer arrhythmia-related artifacts) compared with ECG-gated breath-hold cine imaging in patients without devices but with arrhythmias (13–16). The sensitivity of ECG-gated cine imaging to arrhythmias stems from its acquisition method: Data are collected over multiple heartbeats and combined to reconstruct a single “average” heartbeat dataset. Heart rate variability can cause misregistration in k-space, resulting in ghosting and/or blurring artifacts, possibly resulting in poor or nondiagnostic image quality. In contrast, real-time cine imaging minimizes arrhythmia-induced artifacts because it employs real-time or single-shot (nonrepetitive) acquisition (29), eliminating dependence on heartbeat consistency. To our knowledge, this represents the first evaluation of real-time cine MR image quality specifically in patients with CIEDs with arrhythmias, demonstrating the clinical viability of this approach.

Our previous study established that a 16-fold accelerated Cartesian real-time cine sequence (39.8-msec temporal resolution) provided diagnostically acceptable image quality and accurate left ventricular functional measurements in patients with CIEDs and sinus rhythm, showing strong agreement with standard ECG-gated cine sequence (19). The current study differs from the study by Hong et al (19) in two key aspects. First, although Hong et al evaluated 13 patients with CIEDs and sinus rhythm, we studied 23 patients with CIEDs and arrhythmias—a more clinically challenging study sample. Second, our 32-fold accelerated radial real-time cine sequence (19.3-msec temporal resolution) demonstrated superior image quality compared with the 16-fold accelerated Cartesian real-time cine sequence (39.8-msec temporal resolution).

This study had limitations that warrant further discussion. First, our study sample comprised 23 patients with CIEDs and arrhythmias using real-time cine sequences, and this modest size might limit generalizability. Future studies with larger study samples and different types of arrhythmias are needed to completely validate these findings. Second, we focused on image quality metrics but did not directly compare biventricular volumetric and functional parameters between the radial and Cartesian real-time cine sequences. The inherent variability of cardiac function during arrhythmia presents unique challenges for comparing functional parameters not simultaneously measured (ie, moving targets). A fundamental challenge in the population is the current inability to determine whether observed differences in ventricular function are attributable to the imaging pulse sequence or to the irregularly irregular rhythm. Consequently, we restricted our comparative analysis to image quality metrics. Third, we did not interpret ECG during MRI because of the magnetohydrodynamic effects that distort ECG signals inside MRI scanners (27). Although magnetohydrodynamic effects precluded precise rhythm diagnosis during scanning, the acquired ECG tracings nevertheless enabled quantification of the arrhythmia burden during heart rate variability analysis.

In conclusion, the proposed 32-fold accelerated radial real-time cine pulse sequence provides higher temporal resolution and superior image quality in patients with CIEDs and arrhythmias than the previously introduced 16-fold accelerated Cartesian cine pulse sequence. These findings suggest that this accelerated radial real-time cine approach could enable more accurate cardiac functional assessment in patients with CIEDs and high arrhythmia burden, potentially improving clinical decision-making. Furthermore, the superior image quality and reduced scan time may facilitate broader adoption of real-time cine MRI in this challenging patient population.

## Supplemental Files

Figure S1

Conflicts of Interest
