# Supplementary material for: Highly Accelerated Real-time Cine MRI Pulse Sequence for Cardiac Implantable Electronic Devices and Arrhythmias
Source: Radiol Cardiothorac Imaging. 2025 Nov 13;7(6):e240554. doi: 10.1148/ryct.240554 (PMC12728514; doi:10.1148/ryct.240554)
Supplement: Figure S1 [file ryct240554suppa1.pdf]

Supplemental materials undergo peer review but are not copyedited, and may include typographical errors.

©RSNA, 2025
10.1148/ryct.240554

Supplementary Materials

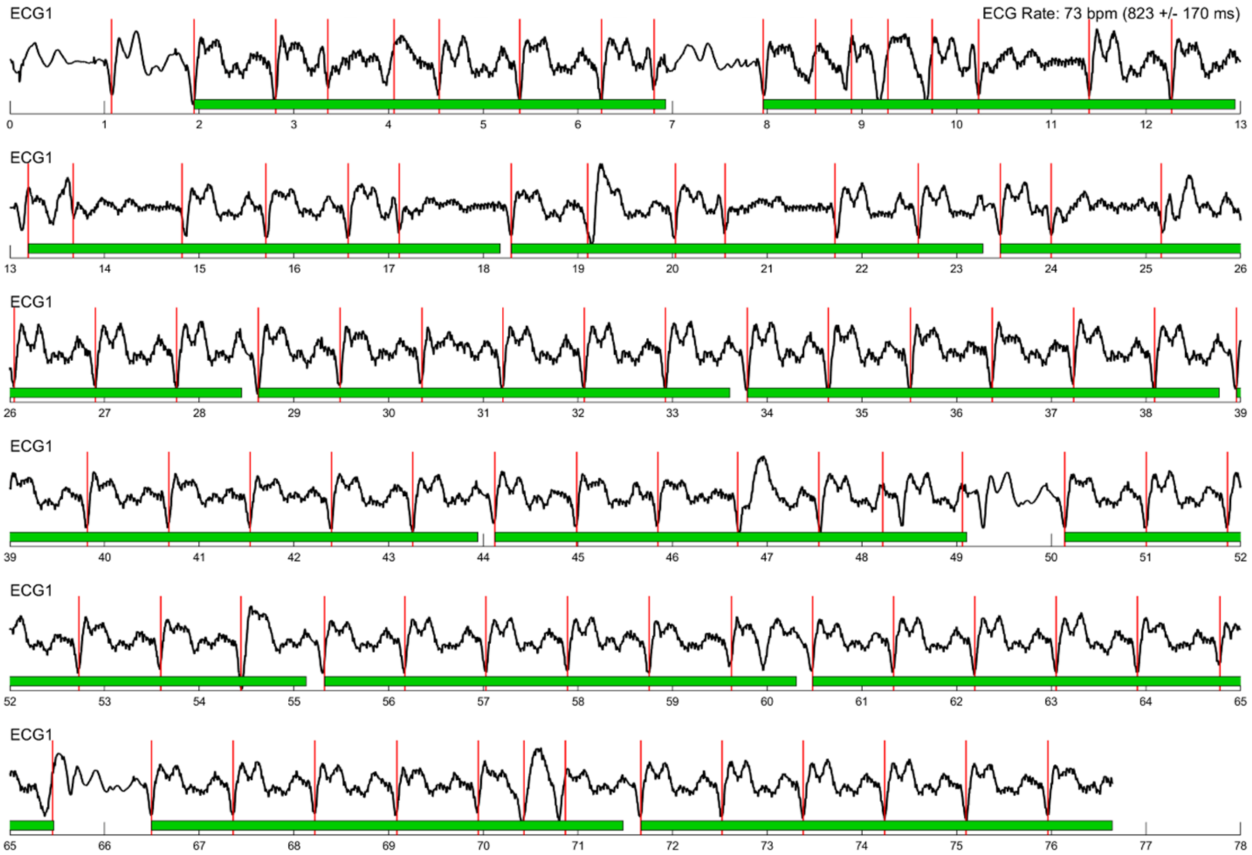

Figure S1: The “ECG” trace of the patient shown in Figure 3, HRV = 20.7%. This information was extracted from the metadata embedded in the raw k-space data. ECG = electrocardiogram, HRV = heart rate variability.
